# Transform-MinER: transforming molecules in enzyme reactions

**DOI:** 10.1093/bioinformatics/bty394

**Published:** 2018-05-14

**Authors:** Jonathan D Tyzack, Antonio J M Ribeiro, Neera Borkakoti, Janet M Thornton

**Affiliations:** EMBL-EBI, Wellcome Genome Campus, Cambridge, UK

## Abstract

**Motivation:**

One goal of synthetic biology is to make new enzymes to generate new products, but identifying the starting enzymes for further investigation is often elusive and relies on expert knowledge, intensive literature searching and trial and error.

**Results:**

We present Transform Molecules in Enzyme Reactions, an online computational tool that transforms query substrate molecules into products using enzyme reactions. The most similar native enzyme reactions for each transformation are found, highlighting those that may be of most interest for enzyme design and directed evolution approaches.

**Availability and implementation:**

https://www.ebi.ac.uk/thornton-srv/transform-miner

## 1 Introduction

One goal of synthetic biology is to design or evolve new enzymes to perform new reactions or generate new products (Renata *et al.*, 2015). However, identifying the most similar enzyme reactions with potential for promiscuous enzyme-substrate interactions (Khersonsky and Tawfik, 2010; Nobeli *et al.*, 2009) to act as start points for further investigation is often challenging, relying on expert knowledge and intensive literature searching.

We present Transform-MinER (Transform Molecules in Enzyme Reactions), an online computational tool that transforms query substrate molecules into products by applying known enzyme reactions at potential reaction centres (RCs) and retrieves the most similar native enzyme reactions for each. It can be used in two modes: (i) Molecule Search identifies potential enzyme transformations acting on a submitted query substrate; and (ii) Path Search attempts to link submitted source and target molecules with enzyme transformations.

## 2 Materials and methods

Transform-MinER is based on three main tasks: Section 2.1 identifies potential RCs in query substrates; Section 2.2 calculates the RC molecular environment (MolEnv) similarity in query and native substrates in order to generate rank ordered lists; and Section 2.3 applies transformations to produce products. The methodology and user interface are described below, with further explanation provided in the ‘About Transform-MinER’ section of the website.

### 2.1 Reaction centres

The data behind Transform-MinER was obtained from the Kyoto Encyclopedia of Genes and Genomes (KEGG) database (containing *c.* 11k enzyme reactions) (Kanehisa *et al.*, 2016) and balanced Reaction (RXN) files were generated for all reactions where molecular structures were available. RCs were defined as atoms that change connectivity, neighbours or stereochemistry and were identified from mapped RXN files after performing atom–atom mapping (Rahman *et al.*, 2014). Canonical SMiles ARbitrary Target Specification (SMARTS) patterns were generated (Landrum, 2018) to represent these RCs, enabling query molecules to be searched for matching fragments.

### 2.2 Similarity of RC MolEnvs

The MolEnv of each RC was defined as all atoms in the substrate outside the RC to a bond depth of 10 bonds and represented using MolPrint2D circular fingerprints (Bender *et al.*, 2004). This enabled Tanimoto similarity scores between query and native RC MolEnvs to be calculated, allowing rank ordered lists of similar enzyme reactions to be generated.

### 2.3 Reaction SMARTS

RDKit Reaction SMARTS patterns (Landrum, 2018) were generated from the mapped RXN files (2.1), describing how each substrate is transformed into the product at each RC. For example, KEGG reaction R02377 involving the oxidation of propan-1-ol to propanal can be expressed as:

[C&X4&H2: 1]-[O&X2&H1: 2]≫([C&X3&H1: 1]=[O&X1&H0: 2])

where &X represents the total number of neighbours, &H represents the number of bound hydrogens and atom identifiers are given after the colon. The Reaction SMARTS can then be applied to potential RCs in a query substrate to generate the products from the transformation.

## 3 User interface

The potential RCs identified in the query substrate, RC MolEnv similarity scores (with hyper-links to the KEGG database) and transformation products are returned to the user in an interactive web application (Django Software Foundation, 2018). This allows a query substrate to be submitted (Molecule Search) from the MarvinSketch plugin (ChemAxon, 2016) along with a target molecule if known (Path Search).

Results are presented to the user using two main interactive views: (i) Path View, with nodes representing molecules and edges representing transformations; and (ii) Molecule View, with interactive shapes representing RCs. A similarity slider allows the user to vary the similarity threshold between the submitted minimum similarity threshold (MinSimThresh) and 1.0 to control the number of transformations presented to the user. When selecting a transformation (an edge in Path View or a simplified Reaction SMARTS embedded in an RC shape in Molecule View) a Similar Enzyme Reactions Data Table is populated showing matching native enzyme reactions and substrates in descending RC MolEnv similarity. Hyperlinks take the user to the associated KEGG reaction and KEGG compound, with the native KEGG compound structure in a hover-over box.

### 3.1 Molecule search

The algorithm uses SMARTS pattern matching to identify fragments in the query substrate that match native RCs, and applies Reaction SMARTS patterns to generate products where query and native RC MolEnv similarity is above MinSimThresh.

Path View shows all transformations radiating from the submitted query substrate (source) which can be selected to populate the Similar Enzymes Reactions Data Table. The edges are labelled with the best RC MolEnv similarity score for that transformation and a hover-over box identifying the most similar KEGG reaction and compound.

Molecule View has hover-over functionality embedded in the RC shapes that reveals simplified Reaction SMARTS patterns describing potential transformations and RC MolEnv similarity scores. Selecting a simplified Reaction SMARTS pattern produces the product by applying the selected transformation at the selected RC, and populates the Similar Enzyme Reactions Data Table. A detailed list of transformations is provided beneath the Interactive Molecule View in an expandable Accordion View.

### 3.2 Path search

If a target molecule has been submitted, Transform-MinER applies transformations iteratively, retaining only those paths that move closer to the target molecule and discarding others. The algorithm prioritizes exploration of paths by taking the product of the transformation-product/target-molecule similarity (calculated using Morgan fingerprints in RDKit) and the highest RC MolEnv similarity, exploring paths with higher combined scores first. The Molecule view is the same as described previously, showing RCs and transformations from the source molecule.

### 3.3 Example

**Fig. 1.**
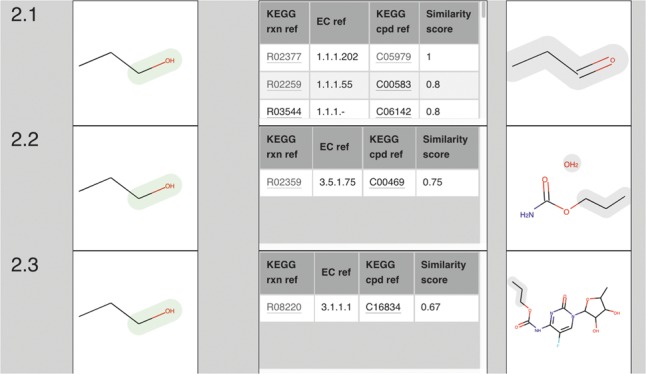
Part of the Accordion View for propan-1-ol with similarity threshold 0.6. For example, 2.1 shows the transformation of propan-1-ol to propanal, with RC MolEnv similarity of 1.0 to KEGG compound C05979 in KEGG reaction R02377 being the best match in the data table. The RC is highlighted in green in the source molecule, the grey highlighting in the product molecule maps to the source molecule

A screenshot of part of the Accordion View obtained when submitting propan-1-ol in a Molecule Search with similarity threshold of 0.6 is shown in Figure 1.

## 4 Conclusions

Transform-MinER provides an interactive way of applying enzyme transformations to query substrates, finding the most similar native enzyme reactions for each, and is complementary to other computational tools for predicting enzyme transformations (Delépine *et al.*, 2018; Moriya *et al.*, 2010). It is anticipated that this tool will help to identify substrates that may show promiscuous activity with enzymes, acting as start points for further development using synthetic biology methods.

## Funding

This work was supported by the European Molecular Biology Laboratory (EMBL).

*Conflict of Interest*: none declared.
